# Ethnic density, social support, and loneliness among Chinese immigrants in Philadelphia

**DOI:** 10.1016/j.wss.2021.100050

**Published:** 2021-07-29

**Authors:** Marilyn Tseng, Emily Walton, Elizabeth Handorf, Carolyn Y. Fang

**Affiliations:** a Department of Kinesiology and Public Health, California Polytechnic State University, San Luis Obispo, CA, USA; b Department of Sociology, Dartmouth College, Hanover, NH, USA;; c Cancer Prevention and Control Program, Fox Chase Cancer Center, Philadelphia, PA, USA

**Keywords:** Ethnic density, Immigrant health, Loneliness, Social support, Family, Friends

## Abstract

Living in more ‘ethnically dense’ areas is thought to promote health, possibly by facilitating social support and a sense of belonging. Because of kin networks and cultural obligations, family relationships may be particularly important for Asian immigrants. Chinese-origin individuals are the largest group of Asian Americans and among the most highly segregated, but the psychosocial benefits of living in Chinese neighborhoods are not established. We examined whether Chinese immigrants in areas of higher ethnic density report more social support from family and friends, and less loneliness. For 606 participants recruited 1/2016–5/2019 throughout the Philadelphia region, residences were linked to American Community Survey 2013–2017 data. Ethnic density, operationalized as percent of Census tract residents who were Chinese, was categorized into quintiles. Family/friend support and loneliness were self-reported, then dichotomized to distinguish high levels of family support, friend support, and loneliness. In logistic regression adjusting for age, sex, and individual- and tract-level socioeconomic characteristics, ethnic density was associated with high family support (odds ratio (OR) 1.85, 95% confidence interval (CI) 1.09, 3.11) for highest vs. lowest ethnic density quintile)) and inversely associated with loneliness (OR 0.31, 95% CI 0.12, 0.79, highest vs. lowest quintile). Our findings support the hypothesis that residents of areas with higher ethnic density report more social support from family and less loneliness. Whether these benefits arise from characteristics of the community overall or from the aggregation of individual assets remains to be clarified but has implications for efforts to develop community resources that would benefit all their residents.

## Introduction

Working from the ‘poverty paradigm’ (Morenoff et al., 2001), the sociological literature theoretically treats highly segregated areas as socioeconomically disadvantaged (Massey and Denton, 1993). Residents of neighborhoods with high racial minority concentration are theorized to have poorer health outcomes because they are separated from resources and opportunities to care for their health (Robert, 1999). Widespread racial and ethnic diversification since the 1965 Immigration and Nationality Act demands that researchers expand our theoretical thinking about residential segregation beyond the poverty paradigm and beyond black and white.

Related (but not equivalent (Massey and Denton, 1988)) to the phenomenon of residential segregation is the ethnic composition of a neighborhood. Recent investigations have demonstrated how living in areas with higher proportions of people of the same ethnicity has also been shown to promote health and well-being – a concept termed the ‘ethnic density effect’ (Becares et al., 2018; Shaw et al., 2012; Walton, 2009). The specific mechanisms linking ethnic density with health are under-explored and may include effects on social capital and health behaviors (Yang et al., 2018). Among Asian and Latinx Americans in particular, living in ethnically dense areas is thought to translate into greater access to culture- and language-specific education and health resources (Walton, 2012; Whitley et al., 2006), and social resources (Das-Munshi et al., 2010) that individuals may draw upon to maintain good health, although the effects often vary among and within ethnic groups (Hong et al., 2014; Walton, 2015; Yang et al., 2018). The current study resolves some of these tensions with our examination of the ways ethnic density relates to the mechanisms of family/friend support and loneliness among Chinese immigrants.

### Ethnic density and US Chinese immigrants

Because the literature on the psychosocial effects of ethnic density has mostly focused on Latinx (Almeida et al., 2009; Shell et al., 2013; Viruell-Fuentes et al., 2013) or African American (Vogt Yuan, 2007) populations, further exploration of this potential link in other immigrant groups would provide empirical insight into its generalizability to different populations. Between 2000 and 2015, the Asian American population increased by 72%, from 11.9 to 20.4 million – the fastest growth rate of any major racial group in the US (López et al., 2017). Individuals of Chinese origin make up the largest group (23%) of Asian Americans (Budiman et al., 2019), and 63% of the Chinese American population is foreign-born (López et al., 2017). Although Chinese Americans have settled across many areas of the country, they are among the most highly segregated Asian Americans (Iceland et al., 2014; Logan and Zhang, 2013), more highly segregated from whites than are Latinx groups (Logan and Zhang, 2013).

For Chinese immigrants, Chinatowns have been seen as spaces of social and economic support (Liu and Geron, 2008; Zhou and Logan, 1991). Traditionally, they have been theoretically seen as centers for family-run businesses that provide ‘an economic basis for stable family life’ (Nee and Wong, 1985). An analysis of more recent Asian American patterns of residence in California supports the continued importance of Chinese immigrant enclaves in helping its residents achieve socioeconomic mobility (Walton, 2015). Previous studies on ethnic density and social resources in Asian Americans examined Asian Americans as a whole (Hong et al., 2014; Walton, 2012). Because Asian Americans are such a widely heterogeneous population (Iceland et al., 2014; Logan and Zhang, 2013; Walton, 2015), the effects of ethnic resources may differ among Asian ethnic groups (Walton, 2015), and by generation/nativity (Becares et al., 2012; Denton et al., 2015; Shell et al., 2013). As a result, the theoretically salubrious social effects for Chinese immigrants of living in Chinese neighborhoods remain unconfirmed.

### Ethnic density and social support

The psychosocial benefits of ethnic density can be conceptualized as a community-level resource – i.e., an attribute of the community as a whole and its social organization (Kawachi et al., 2008) – or as the aggregation of ‘resources accrued at the individual level as a result of one’s membership of social networks’ (Kim et al., 2013). Evidence of these presumed psychosocial benefits is mixed, depending on whether they are conceptualized as community- or individual-level characteristics. Previous studies have shown no association (Becares and Nazroo, 2013; Walton, 2012) or even an inverse association (Almeida et al., 2009; Osypuk et al., 2009) between ethnic density and social cohesion, a community-level characteristic representing residents’ perceived levels of trust among their neighbors. In the National Latino and Asian American Study (Hong et al., 2014), ethnic density was positively associated with social cohesion among Latinos but negatively among Asians.

Studies that examined ethnic density in relation to individual perceptions of their social support and social networks – i.e., an individual asset – are more consistent. In two studies conducted among Latinos in Chicago, residents of areas with higher ethnic density reported more family and friends in the neighborhood (Almeida et al., 2009), had larger and more diverse social networks, and more frequently got together or talked/emailed with friends, neighbors, and relatives (Viruell-Fuentes et al., 2013). Other studies also observed associations between ethnic density and social support, variously measured, among people of Mexican-descent in Texas (Shell et al., 2013), African Americans in Illinois (Vogt Yuan, 2007), and Bangladeshis in England (Das-Munshi et al., 2010). Overall, existing but limited evidence suggests that ethnic density exerts positive effects through the aggregation of individual resources, possibly individually held social relationships that bring a sense of integration and belonging.

The availability and sources of social support are especially important for racial/ethnic minorities given previous evidence suggesting that their social networks tend to be smaller than those of non-Hispanic Whites (McPherson et al., 2006; Park et al., 2015), and also more reliant on family (Park et al., 2015). Family relationships may be particularly central to social relationships and feelings of belonging among Asian Americans because of traditions of familial obligation and duty (Zhang and Ta, 2009) as well as the threat of ‘losing face’ from sharing personal problems in less intimate social networks (Cheng et al., 2016). Findings among Asian participants in the National Latino and Asian American Study also suggest the primacy of family support in reducing psychological distress. In one analysis, acculturative stress was significantly inversely associated with family support, but not with friend support (Singh et al., 2015). In a separate analysis, spousal support was more important than more distal forms of social support as a buffer against unfair treatment (Rollock and Lui, 2016), again suggesting that familial relationships play a greater role than other sources of support for some Asian American populations.

### Ethnic density and loneliness

A lack of meaningful social relationships may contribute to social isolation (an objective measure of lack of interaction with others) and loneliness (the negative, subjective experience of a perceived deficiency in the quality or quantity of one’s social relationships) (Leigh-Hunt et al., 2017; Park et al., 2020). Loneliness itself is a risk factor for poorer health, both mental and physical (Leigh-Hunt et al., 2017). Qualitative studies among immigrants indicate that the absence of family and close friends, less intimate social relationships with neighbors, and an uncertain sense of belonging all contribute to feelings of loneliness (Hurtado-de-Mendoza et al., 2014; Li et al., 2018). These findings are consistent with quantitative studies showing higher levels of loneliness among immigrants than non-immigrants (Ten Kate et al., 2020; van Tilburg and Fokkema, 2018).

Characteristics of the neighborhood environment may be important. Among young adults in Great Britain, perceiving one’s neighborhood as having lower collective efficacy was associated with greater loneliness, although objectively measured characteristics of the neighborhood such as socioeconomic status and crime were not (Matthews et al., 2019). In contrast, neighborhood socioeconomic deprivation was associated with loneliness in nationally representative data from Denmark; residents of deprived neighborhoods had 50% higher odds of loneliness compared to the general population, even with adjustment for differences in individual-level education and employment status (Algren et al., 2020). To our knowledge, no previous studies have examined whether ethnic density is associated with feeling less lonely.

The objective of this analysis was to examine the association of ethnic density with social support from family and friends and with loneliness in a sample of Chinese immigrants in the Philadelphia region. We hypothesized that Chinese immigrants residing in areas of higher ethnic density would report more social support and less loneliness than immigrants living in areas of lower ethnic density.

## Materials and methods

### Study sample and procedures

Between January 2016 and May 2019, research staff recruited 650 Chinese immigrants living in the Philadelphia region to participate in the study. We focused on Chinese immigrants given evidence of their substantial experience of post-migration and acculturative stressors, particularly in the form of feelings of cultural disorientation, lack of integration, and homesickness (Ying et al., 2012). Community outreach and recruitment efforts included attending community organization meetings, activities, and events, and enlisting the support of key organizations, churches, and leadership contacts in areas of Chinese concentration. Eligibility criteria were Chinese heritage, age 35–65 years, and immigration from Asia as an adult (age 18+ years). Exclusion criteria were: self-reported history of diabetes, cancer, auto-immune disorders, or HIV infection; use of medications relevant to study outcomes (e.g., anti-inflammatory medications); current pregnancy or breastfeeding; and inability to provide informed consent. Participants received $60 for their participation. The study was approved by the Fox Chase Cancer Center Institutional Review Board, and all participants gave their written, informed consent to participate. Participants received all contact materials, including consent forms and questionnaires, in both Chinese and English.

### Measures

Interviewers fluent in Chinese conducted detailed interviews, either in person or over the telephone, in the appropriate dialect (Mandarin or Cantonese). We assessed sociodemographic characteristics including age, gender, marital status, highest level of education, participant’s usual occupation, and the usual occupation of their spouse. Usual occupation was categorized as one of three main occupational categories: blue collar, service, or white collar (Asfar et al., 2016). Self-employment was categorized with white collar occupations. When participants reported their own and their spouse’s occupation, they were assigned an occupational category based on the higher of the two. We measured acculturation using an abridged 11-item version of the General Ethnicity Questionnaire - American version (GEQA) (Tsai et al., 2000), which assesses the respondent’s English language use and exposure to or engagement with American people, culture, and activities (e.g., ‘I celebrate American holidays’, ‘At home, I eat American food’), with a minimum of 1.0 (least acculturated) and a possible maximum score of 5.0 (most acculturated). The scale demonstrated high internal reliability in the present sample (Cronbach’s α=0.86) and in prior studies (Tseng and Fang, 2014).

For social support, we used the 15-item Provisions of Social Relations (PSR) scale (Turner et al., 1983) to capture respondents’ perceptions of five types of ‘provisions’ – i.e., assistance or resources – obtainable from social relationships: attachment (emotional closeness), social integration (sense of belonging), reassurance of worth (recognition or validation of one’s value and competence), reliable alliance (perceived assurance of assistance from others when needed), and guidance (having others who can provide advice or information) (Weiss and Rubin, 1974). The PSR has good psychometric properties (Turner et al., 1983) and has been used successfully with Chinese (Shen and Takeuchi, 2001) and Korean immigrants (Noh and Avison, 1996). The scale is comprised of two subscales: a nine-item Friend Support subscale and a six-item Family Support subscale. The nine items assessing provisions from friend relationships include statements such as, ‘I feel very close to some of my friends,’ and ‘When I’m with my friends I feel completely able to relax and be myself’. The remaining six items of the PSR assess provisions from relationships with family (e.g., ‘I know my family will stand by me,’ ‘My family lets me know they think I’m a worthwhile person’). Response options range from 1 (very much) to 5 (not at all). Scores for friend and family support were computed as the sum over the relevant nine and six items, respectively, with a higher score representing greater perceived provisions. In the present sample, both the friend and family support subscales demonstrated high internal reliability (Cronbach’s α=0.89 for each subscale).

Loneliness was assessed using the validated, three-item University of California – Los Angeles loneliness scale, which captures perceptions about lack of companionship, feeling left out, and feeling isolated (Cornwell and Waite, 2009; Hughes et al., 2004). The three-item scale demonstrated high reliability in the present sample (α=0.87). Scores were computed by standardizing and summing over the three items. This scale has been used extensively in research (Cornwell and Waite, 2009; National Academies of Sciences E, and Medicine 2020; Park et al., 2020; Steptoe et al., 2013), including in Chinese populations (Liu et al., 2019).

### Census tract characteristics

Residences were geocoded and linked to American Community Survey 2013–2017 data. Ethnic density was operationalized as the proportion of Census tract residents who were Chinese. We also examined other Census tract-level variables commonly used as indicators of socioeconomic disadvantage (Krieger et al., 1997) as potential confounders. These included proportion of adults age 25 and older with a college degree; percent of occupied housing units that were owner-occupied; percent of adults age 18–64 years living in poverty; and median household income.

### Statistical analysis

Of the 650 participants recruited into the study, one withdrew, two did not provide a residential address, and seven lived in a state other than Pennsylvania. An additional 34 participants were excluded for missing covariate data, leaving a sample of n=606 for this analysis.

We used analysis-of-variance to evaluate bivariate associations of covariates with quintiles of ethnic density. In multivariate analyses, we ran logistic regression models to estimate odds ratios (OR) with corresponding 95% confidence intervals (CI) using Generalized Estimating Equations with an exchangeable correlation matrix to account for clustering within Census tracts. Because its distribution was highly skewed with most Census tracts clustered at low levels, ethnic density, the independent variable of interest, was primarily modeled as nominally coded quintiles. Trend p-values for quintiles were estimated by modeling the median ethnic density for each quintile as a continuous predictor variable. Additional models including linear and quadratic terms for ethnic density showed no significant effects for square terms; results for the linear, continuous ethnic density variable are presented.

We modeled the outcome variables of family support, friend support, and loneliness in separate analyses, dichotomized depending on their distributions. Distributions for all three outcome variables were highly skewed. Friend support clustered towards higher scores. We defined high friend support as a score in the highest quintile; alternative cutpoints (i.e., highest tertile or quartile) produced similar but less pronounced results. Family support was also clustered towards higher scores, with about a third (35%) of all respondents reporting the highest possible score (30) on this scale; therefore, we defined high family support as having a score of 30. In contrast, loneliness clustered towards lower scores, with almost 70% of respondents having a z-score <0. We defined loneliness as a z-score >1.5 for the three-item scale, where a natural break in the distribution was apparent; a less conservative cutpoint (z-score >1.0) produced similar but less pronounced results. Additionally, findings were similar when we modelled the three outcome variables as continuous variables, both untransformed and transformed for normality. For easier interpretability, we present findings based on the dichotomized variables only. Findings from the additional analyses are available in the Supplemental Table.

Variables expected to be associated with ethnic density and/or the outcome variables based on prior research were included as potential confounders in fully adjusted models. These were age (continuous years), sex, marital status (married or not), education level (<8 years, 8–11 years, high school graduate, Bachelor’s degree or higher), occupational category (blue collar, service, or white collar occupation), acculturation level (continuous GEQA score), percent of adults in the Census tract with a college degree, median household income of the Census tract, percent of adults in poverty in the Census tract, and percent of homes in the Census tract that were owner-occupied.

In additional analyses we examined associations of family and friend support with loneliness as the outcome variable. We also evaluated family and friend support as potential mediators by examining whether the association of ethnic density with loneliness persisted with adjustment for those variables.

All analyses were conducted using SAS (version 9.4, 2013, SAS Institute, Inc., Cary, NC).

### Results

**Fig. 1.**
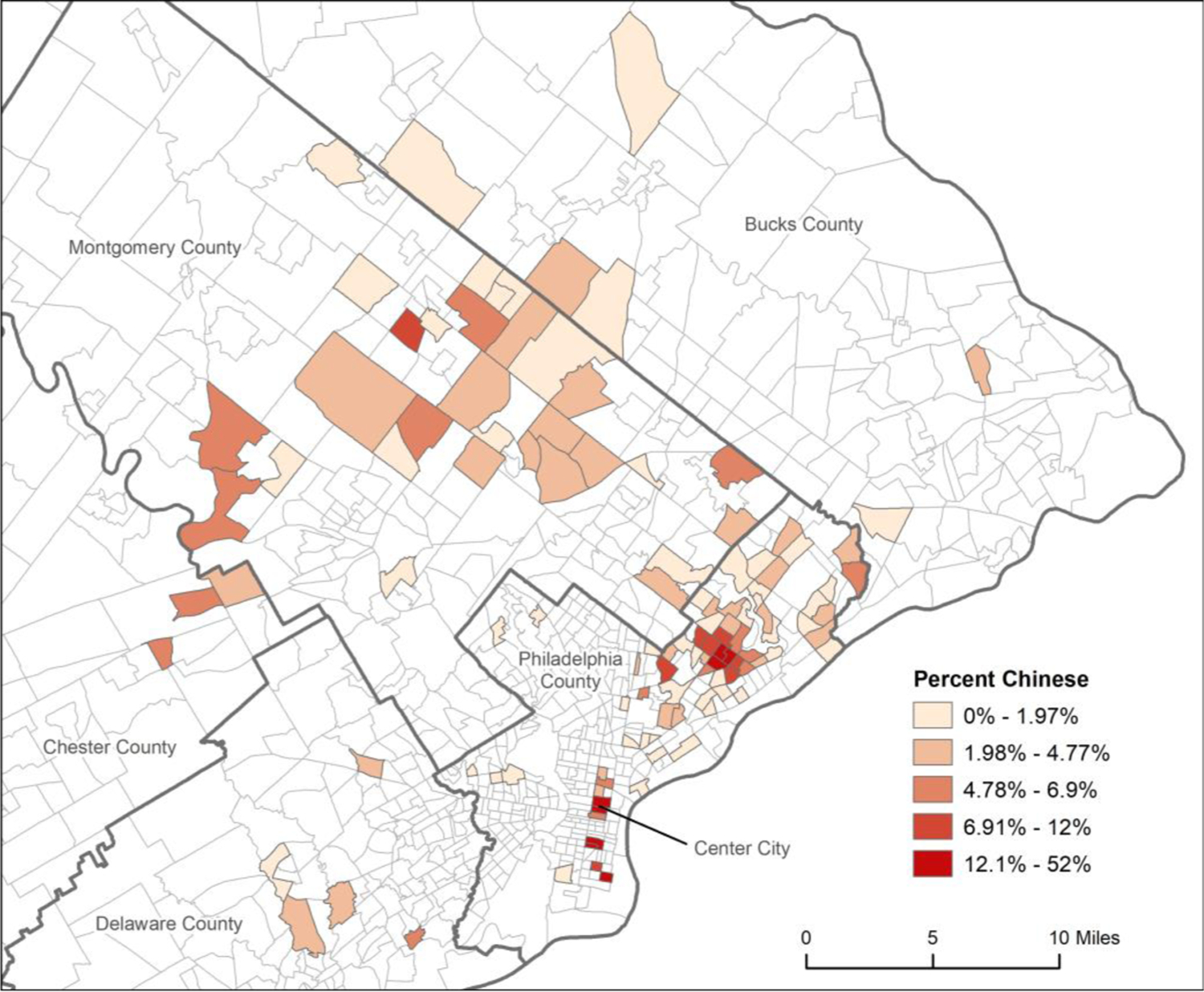
Census tracts (n=120) represented by 606 Chinese immigrant participants recruited January 2016 and May 2019 from the Philadelphia, PA region.

Participants in the sample resided in 120 different Census tracts in the Philadelphia region (Fig. 1), with a mean (SD) population of 4,962 (1,655) (Table 1). The proportion of Chinese residents in these 120 Census tracts ranged from 0 to 52.0%, with average numbers of Chinese residents ranging from 28 to 942 in the first to fifth quintiles of ethnic density. Significant differences in other characteristics across tract quintiles were driven primarily by higher socioeconomic indicator values in the second quintile, where an average of 42% of adult residents in those tracts had a college degree and median household income averaged >$81,000, and lower values in the fourth and fifth quintiles, where >20% of adult census tract residents were in poverty (Table 1).

In our sample of 606 participants (Table 2), mean (SD) age was 51.0 (7.6) years, 57.7% were female, and 89.0% were married. Our sample showed a range of education levels, with 33% having <8 years of education and 16% having at least a college degree, as well as a range of acculturation levels (mean 2.79, range 1.0–4.82 out of a maximum score of 5). Participants residing in Census tracts with the highest ethnic density were less likely to be college-educated and to report a white-collar occupation as their usual occupation, had slightly lower acculturation scores, and were less likely to be classified as lonely (Table 2).

Individuals with high family support were more likely to report high friend support than those who reported less family support (43% vs. 12%, p<0.0001). Individuals with high family support also reported less loneliness; only 7% of those with high family support were categorized as lonely, compared with 19% among those with less family support (p<0.0001). Similarly, 5% of those with high friend support were categorized as lonely, compared to 18% among those with less friend support (p=0.0003).

In multivariate logistic regression models (Table 3), living in Census tracts with higher ethnic density was associated with high family support (OR 1.85 (95% CI 1.09, 3.11) for ethnic density quintile 5 vs. quintile 1, trend p=0.09), although a gradient across quintiles of ethnic density was not apparent. Residents in the highest quintile for ethnic density were also significantly less likely to be lonely (OR 0.31 (95% CI 0.12, 0.79) for quintile 5 vs. quintile 1, trend p=0.02). The inverse association between ethnic density and loneliness persisted (OR 0.36 (95% CI 0.14, 0.92) for quintile 5 vs. quintile 1, trend p=0.04) with adjustment for both family and friend support, both of which were also significantly inversely associated with loneliness in multivariate models (OR 0.36 (95% CI 0.17, 0.73) for high vs. lower family support, and 0.40 (95% CI 0.16, 0.98) for high vs. lower friend support) (Table 4).

## Discussion

The primary finding in this sample of Chinese immigrants was that compared to those living in areas of lower ethnic density, residents of higher ethnic density areas reported more social support, particularly from family, and less loneliness. Assessments of the adequacy of social support from friends did not differ by neighborhood ethnic composition. The observed associations were apparent even with adjustment for potential confounders including Census tract-level poverty. Further, participants who reported high levels of family and friend support were significantly less likely to be lonely, although this did not explain the inverse association between ethnic density and loneliness.

Ethnically dense neighborhoods may provide the opportunities and gathering spaces to nurture social networks and ties, such as senior centers and churches (Li et al., 2018; Syed et al., 2017). Previous studies in Latino, African American, and Asian immigrant populations have observed associations of higher ethnic density with more extensive social networks and ties, better social integration, and greater perceived social support (Almeida et al., 2009; Das-Munshi et al., 2010; Shell et al., 2013; Viruell-Fuentes et al., 2013; Vogt Yuan, 2007). Among studies that attempted to parse the specific types of social support associated with ethnic density, Viruell-Fuentes et al. (Viruell-Fuentes et al., 2013) found that ethnic density was associated with social integration and social networks among Latinos in Chicago, but not with perceived availability of instrumental or informational support. Among Bangladeshis in England (Das-Munshi et al., 2010; Das-Munshi et al., 2012), ethnic density was associated with practical and negative social support and not with confiding or emotional support; however, the measure of social support focused only on perceived support from the one person the respondent reported being closest to (Das-Munshi et al., 2010; Das-Munshi et al., 2012). We identified an association of ethnic density specifically with family rather than friend support – a distinction not examined in prior studies. Because family ties likely exist prior to choosing to live in a given neighborhood, our findings suggest that the association of ethnic density with social support is the result of the aggregation of individual resources, rather than a result of social networks developed by residing in an ethnically dense neighborhood. Differences in family size and structure may produce different findings – a possibility that could not be assessed in this study. Thus, the finding warrants confirmation in other populations and settings.

Ours is the first study to our knowledge to show that residents of more ethnically dense areas are less likely to be lonely. In our sample of Chinese immigrants, the association was not explainable by higher levels of social support, suggesting that ethnically dense areas also protect against loneliness through other means. Qualitative studies point to the importance of a sense of belonging and of trusting relationships with neighbors to reduce feelings of isolation and loneliness (Hurta-do-de-Mendoza et al., 2014; Li et al., 2018). In addition, neighborhoods in which physical spaces incorporate Chinese cultural symbols may increase Chinese immigrants’ sense of belonging (Hsu, 2014; Syed et al., 2017). Based on research conducted among elderly Chinese women living in Montreal’s Chinatown, Hsu (Hsu, 2014) surmises, ‘Home […] is where one can make oneself understood and follow others’ reasoning without long explanations or extra efforts. There is less interruption or disturbance in communication […]. The sense of belonging is emotional, and integration is subjective because home is where one feels safely anchored and from which one comfortably navigates.’

Worth noting is that associations were apparent for our sample of Chinese immigrants even though ethnic density was based on the proportion of all Census tract residents who were Chinese regardless of place of birth. Possibly, social resources resulting from higher ethnic density stem specifically from the Chinese immigrant population, which in Philadelphia makes up 60% of the Philadelphia Chinese population (Social Explorer 2020). It may also be that immigrant and non-immigrant Chinese residents in Philadelphia share many of the same social structures in their neighborhoods, including cultural organizations, networks, and other resources.

The associations of ethnic density with family support and loneliness that we observed here warrant confirmation in other racial/ethnic minority populations. Whereas ethnic density was associated with social support in studies among Latinos in Chicago (Almeida et al., 2009; Viruell-Fuentes et al., 2013) and Texas (Shell et al., 2013), in another study conducted in Illinois (Vogt Yuan, 2007), it was associated with social support only for African American and not for Hispanic participants. In the study in England, ethnic density was associated with social support among Bangladeshis (Das-Munshi et al., 2010; Das-Munshi et al., 2012) but not among other ethnic minority groups, which included Indians, Pakistanis, and Black Caribbeans. Inconsistencies across studies may arise from differences not only in measures of social support, but also in the different socioeconomic contexts and residential patterns of different ethnic groups. Some communities may have better resources to support extended family living or other cooperative arrangements, or more cultural institutions and organizations to support structured and successful social networks.

Besides examining the generalizability of these results to other populations and settings, we suggest three avenues for future investigation to build on this work. First, additional research is needed to address the critical question of whether the presumed social benefits of ethnic density are due to qualities of the community as a whole, or to the resources that individual residents have, regardless of where they live. The current findings provide some support for the latter possibility but included no measures of community-level resources. Second, future studies should clarify how living in an ethnically dense neighborhood might facilitate the kinds of social relationships that provide meaningful levels and types of social support; such studies should also consider the extent to which immigrants access social resources outside of their residential neighborhoods. Finally, we suggest exploring other mechanisms besides social support by which ethnic density might reduce loneliness. The public presence of cultural symbols is an example of a community characteristic that might convey respect and contribute to a greater sense of belonging for its residents (Walton, 2016).

As this was a cross-sectional analysis, we are unable to establish that residence in areas of higher ethnic density preceded participants’ feelings regarding social support and loneliness; it remains possible that participants who perceived greater social support and less loneliness chose to live in neighborhoods of higher ethnic density. Collecting information on participants’ reasons for moving into a neighborhood in future work will provide a potential approach to address this possibility of residential selection bias. Also worth noting is that recruitment and data collection took place over three years, during which sociopolitical contexts may have changed. Further, participant recruitment through community organizations and activities may have resulted in underrepresentation of socially isolated individuals in our sample. The underrepresentation is likely to be non-differential across neighborhoods, attenuating observed measures of association, but it may affect generalizability of results. Another limitation is that Census tracts may not correspond to how residents perceive their actual ‘neighborhoods’, and results may differ with the use of different boundaries or geographic levels. However, the resulting error in estimating ethnic density is unlikely to have been differential and thus to have produced the observed associations.

The relatively low level of ethnic density in our sample is also a possible limitation. Most participants in our study were living in areas that were predominantly non-Chinese, as is typical for Asian ethnic neighborhoods given their low proportions in the US population. Notably, the associations of ethnic density with perceived loneliness and social support were still observed despite this limitation, suggesting that a very high percentage of co-ethnic residents is not necessary to observe a beneficial effect among immigrants. Finally, our data did not capture our participants’ activity space, or whether our participants’ sources of social support lived in the same neighborhood. Future work should address this issue more directly by collecting information on the locations of the social resources both accessed by and accessible to participants.

In sum, we found evidence to support that Chinese immigrants living in areas of higher ethnic density report more support from family and less loneliness. A clearer understanding of how ethnic density relates to psychosocial outcomes has implications for strategies to support the development of community resources that will help all of its residents thrive, not only those with access to resources through their own networks.

## Supplementary Material

Supplementary Table

## Figures and Tables

**Table 1:** Descriptive characteristics of 120 Census tracts represented in sample of 606 Chinese immigrant participants recruited January 2016 and May 2019 from the Philadelphia, PA region.

	All	Ethnic density quintiles^a^					p-value
		1 0–<1.97%	2 1.97–<4.77%	3 4.77–<6.9%	4 6.9–<12.0%	5 12.0–<52.0%	
N	120	54	34	16	8	8	
Total population	4962 (1655)	4747 (1491)	5270 (1749)	4637 (1959)	5648 (1237)	5073 (1991)	0.39
Total Chinese population	197 (285)	28 (28)	163 (67)	271 (127)	589 (180)	942 (484)	<0.0001
% of residents who are Chinese	3.9 (6.2)	0.6 (0.6)	3.1 (0.7)	5.8 (0.7)	10.3 (1.1)	20.3 (13.9)	<0.0001
% adults age 25+ years with a college degree	34.9 (20.7)	31.7 (18.6)	42.2 (22.0)	38.9 (24.0)	22.0 (13.8)	30.4 (19.8)	0.04
Median household income	66,706 (34,547)	61,491 (30,767)	81,122 (38,707)	75,149 (38,558)	45,461 (17,558)	45,000 (11,356)	0.005
% of occupied housing units that are owner occupied	65.1 (20.1)	62.3 (19.22)	73.9 (18.2)	66.7 (24.7)	59.6 (11.7)	48.4 (16.3)	0.006
% adults age 18–64 years in poverty	14.1 (13.1)	14.3 (14.6)	9.9 (9.6)	13.5 (12.4)	21.9 (11.9)	23.1 (11.9)	0.04

a Quintile cutpoints were based on number of participants, not number of census tracts.

b P-values from analysis-of-variance to evaluate census tract characteristics across ethnic density quintiles.

**Table 2:** Descriptive characteristics of sample of 606 Chinese immigrant participants recruited January 2016 and May 2019 from the Philadelphia, PA region.

	All	Ethnic density quintiles					p-value^a^
		1 0–<1.97%	2 1.97–<4.77%	3 4.77–<6.9%	4 6.9–<12.0%	5 12.0–<52.0%	
N	606	121	120	123	112	130	
Mean (SD) age (y)	51.0 (7.6)	50.8 (8.1)	49.6 (7.6)	51.7 (7.6)	51.2 (7.4)	51.7 (7.4)	0.19
% female	57.7	54.6	59.2	55.3	58.9	59.2	0.52
% married	89.0	88.4	88.3	92.7	87.5	86.9	0.66
Education (%)							**<0.0001**
<8 years	33.2	30.6	13.3	34.2	55.4	33.1	
8–11 years	25.0	22.3	26.7	22.8	20.5	32.3	
HS graduate, <Bachelor’s	25.4	24.8	24.2	25.2	20.5	31.5	
Bachelor’s degree or higher	16.4	22.3	35.8	17.9	3.6	3.1	
Occupational category							**<0.0001**
Blue collar	27.7	15.7	23.3	22.8	46.4	31.5	
Service	39.5	43.0	18.3	47.2	42.9	48.5	
White collar, self-employed	32.7	41.3	58.3	30.1	10.7	20.0	
GEQA score	2.79 (0.68)	2.91 (0.57)	3.04 (0.69)	2.79 (0.71)	2.52 (0.62)	2.74 (0.67)	**<0.0001**
High friend support^b^	22.4	17.4	26.7	23.6	17.9	26.2	0.42
High family support^b^	34.8	26.5	40.0	39.0	35.7	33.1	0.53
Loneliness^c^	15.0	22.3	16.7	12.2	16.1	8.5	**0.005**

a P-values from analysis-of-variance to evaluate bivariate associations of covariates with quintiles of ethnic density.

b High friend support was defined as being in highest quintile based on nine items in the Provisions of Social Relations scale capturing provisions from relationships with friends. High family support was defined as having the highest score (30) for six items capturing provisions from relationships with family.

c Loneliness was defined as having a z-score >1.5 based on three questions on perceived frequency of lack of companionship, feeling left out, and feeling isolated.

**Table 3:** Adjusted^a^ odds ratios (95% confidence intervals) for associations of ethnic density quintiles with high family support, high friend support, and loneliness, among 606 Chinese immigrant participants recruited January 2016 and May 2019 from the Philadelphia, PA region.

	Quintiles of ethnic density						Continuous ethnic density
	1	2	3	4	5	Trend p-value^b^	
High friend support^d^	1.0	1.61 (0.77, 3.34)	1.39 (0.64, 3.01)	1.02 (0.52, 2.00)	1.66 (0.96, 2.89)	0.15	1.11 (95% CI 0.94, 1.32)
High family support^d^	1.0	**2.04** **(1.10, 3.78)**	**2.08** **(1.20, 3.60)**	**2.54** **(1.33, 4.83)**	**1.85** **(1.09, 3.11)**	0.09	1.15 (95% CI 0.96, 1.39)
Loneliness^e^	1.0	0.70 (0.34, 1.45)	0.47 (0.16, 1.40)	0.70 (0.31, 1.56)	**0.31** **(0.12, 0.79)**	**0.02**	**0.58** **(95% CI 0.37, 0.91)**

a All models were adjusted for age (continuous years), sex, marital status (married or not), education level (<8 years, 8–11 years, high school graduate, Bachelor’s degree or higher), occupational category (blue collar, service, or white collar occupation), acculturation level (continuous score), percent of adults in the Census tract with a college degree, median household income of the Census tract, percent of adults in poverty in the Census tract, and percent of homes in the Census tract that were owner-occupied.

b P-values for trend were estimated by modeling median value for each quintile of ethnic density.

c ORs for continuous ethnic density are per 10% change in ethnic density.

d High friend support was defined as being in highest quintile based on nine items in the Provisions of Social Relations scale capturing provisions from relationships with friends. High family support was defined as having the highest score (30) for six items capturing provisions from relationships with family.

e Loneliness was defined as having a z-score >1.5 based on three questions on perceived frequency of lack of companionship, feeling left out, and feeling isolated.

**Table 4:** Adjusted odds ratios (95% confidence intervals) for associations with loneliness^a^ as outcome, among 606 Chinese immigrant participants recruited January 2016 and May 2019 from the Philadelphia, PA region.

	Model 1^b^	Model 2^b^	Model 3^c^	Model 4^d^
High family support^e^	0.29 (0.15, 0.57) 0.0002	–	0.36 (0.18, 0.71) 0.003	0.36 (0.17, 0.73) 0.005
High friend support^e^	–	0.29 (0.13, 0.64) 0.002	0.41 (0.18, 0.97) 0.04	0.40 (0.16, 0.98) 0.04
Ethnic density quintile	–	–	–	
1				1.00 (ref)
2				0.79 (0.32, 1.94)
3				0.51 (0.17, 1.52)
4				0.80 (0.36, 1.81)
5				0.36 (0.14, 0.92)
Trend p-value				0.04

a Loneliness was defined as having a z-score >1.5 based on three questions on perceived frequency of lack of companionship, feeling left out, and feeling isolated.

b Adjusted for age (continuous years), sex, marital status (married or not), education level (<8 years, 8–11 years, high school graduate, Bachelor’s degree or higher), occupational category (blue collar, service, or white collar occupation), acculturation level (continuous score), percent of adults in the Census tract with a college degree, median household income of the Census tract, percent of adults in poverty in the Census tract, and percent of homes in the Census tract that were owner-occupied.

c With additional mutual adjustment for family and friend support

d Includes all variables in Model 1, family and friend support, and ethnic density quintiles.

e High friend support was defined as being in highest quintile based on nine items in the Provisions of Social Relations scale capturing provisions from relationships with friends. High family support was defined as having the highest score (30) for six items capturing provisions from relationships with family.
