# Supplementary material for: Ethnic density, social support, and loneliness among Chinese immigrants in Philadelphia
Source: Wellbeing Space Soc. Author manuscript; Available in PMC 2021 Sep 7. (PMC8423378; doi:10.1016/j.wss.2021.100050)
Supplement: Supplementary Table [file NIHMS1732165-supplement-Supplementary_Table.docx]

Supplemental Table. Adjusted^a^ associations of ethnic density quintiles with loneliness, family support, and friend support, among 606 Chinese immigrant participants recruited January 2016 and May 2019 from the Philadelphia, PA region. Odds ratios (with 95% confidence intervals) are based on alternative cutpoints used to dichotomize loneliness and friend support for logistic regression analyses. Beta estimates (with standard errors) are from linear regression models with continuous outcome variables, both untransformed and transformed for normality.

|  | Ethnic density | | | | |
| --- | --- | --- | --- | --- | --- |
| Outcome variable | Quintile 1 | Quintile 2 | Quintile 3 | Quintile 4 | Quintile 5 |
| Loneliness |  |  |  |  |  |
| **Dichotomous** |  |  |  |  |  |
| Upper 30% | 1.0 | 0.74 (0.40, 1.37) | 0.74 (0.34, 1.60) | 0.63 (0.31, 1.29) | **0.54 (0.27, 1.10)** |
| Trend p-value | **0.10** | | | | |
| **Continuous** |  |  |  |  |  |
| Untransformed | Ref | -0.15 (0.14) | -0.21 (0.18) | -0.14 (0.16) | **-0.30 (0.14)** |
| p-value |  | 0.28 | 0.24 | 0.39 | **0.04** |
| Trend p-value | **0.045** | | | | |
| Transformed | Ref | -0.42 (0.40) | -0.47 (0.50) | -0.57 (0.47) | -0.79 (0.44) |
| p-value |  | 0.29 | 0.35 | 0.23 | 0.07 |
| Trend p-value | 0.08 | | | | |
| Friend support |  |  |  |  |  |
| **Dichotomous** |  |  |  |  |  |
| Highest tertile | 1.0 | 1.39 (0.79, 2.42) | 1.18 (0.54, 2.55) | 0.85 (0.51, 1.39) | 1.26 (0.84, 1.90) |
| Trend p-value | 0.64 | | | | |
| Highest quartile | 1.0 | 1.45 (0.82, 2.56) | 1.54 (0.93, 2.56) | 1.02 (0.62, 1.70) | 1.60 (1.07, 2.37) |
| Trend p-value | 0.009 | | | | |
| **Continuous** |  |  |  |  |  |
| Untransformed | Ref | 1.17 (0.86) | 0.34 (1.11) | 0.20 (0.79) | 1.24 (0.66) |
| p-value |  | 0.17 | 0.76 | 0.80 | 0.06 |
| Trend p-value | 0.13 | | | | |
| Transformed | Ref | 87.4 (59.8) | 30.1 (76.7) | 13.3 (53.6) | 85.3 (43.8) |
| p-value |  | 0.14 | 0.69 | 0.80 | 0.052 |
| Trend p-value | 0.14 | | | | |
| Family support |  |  |  |  |  |
| **Continuous** |  |  |  |  |  |
| Untransformed | Ref | 1.04 (0.63) | 1.19 (0.59) | 1.79 (0.61) | **1.36 (0.57)** |
| p-value |  | 0.10 | 0.04 | 0.003 | **0.02** |
| Trend p-value | **0.03** | | | | |
| Transformed | Ref | -0.31 (0.14) | -0.32 (0.01) | -0.42 (0.003) | -0.31 (0.12) |
| p-value |  | 0.03 | 0.01 | 0.003 | 0.009 |
| Trend p-value | **0.04** | | | | |

^a^ All models were adjusted for age, sex, marital status (married or not), education, occupational class (blue collar, service, or white collar occupation), acculturation, and neighborhood-level education, median household income of the Census tract, percent of adults in poverty in the Census tract, and percent of homes in the Census tract that were owner-occupied.
